# Unique Presentation of Refracture of the Distal Radius With Volar Plate Bending in an Elderly Patient

**DOI:** 10.7759/cureus.71803

**Published:** 2024-10-18

**Authors:** Mamoun Belkebir Mrani, Pierre Pirlot, Serge Ayong

**Affiliations:** 1 Orthopedics and Traumatology, Clinique Saint Pierre Ottignies, Ottignies, BEL

**Keywords:** bended volar plate, hardware failure, ortho-geriatric, radius refracture, wrist fractures

## Abstract

This case report presents the unique case of an 80-year-old woman who sustained a refracture of the distal radius following a fall, resulting in a rare instance of volar plate bending. The patient had undergone osteosynthesis with a titanium volar plate 2.5 years earlier, which had fully healed. Radiographs showed a 50° dorsal angulation of the previously implanted plate, necessitating surgical revision. A modified Henry approach was used to remove the bent plate and implant a new one. While literature documents two prior cases of volar plate bending, both involved older plate designs. This report is the first to describe bending in a modern titanium volar plate, making it a rare and significant contribution to current knowledge on implant failure.

## Introduction

Distal radius fractures are common, particularly in the elderly population, and are often treated with volar plate fixation. Volar plates are known for providing stable fixation and favorable outcomes, even in osteoporotic bones [1]. However, the failure of these plates is rare, especially in modern systems using advanced titanium designs. This case report presents an 80-year-old female patient who sustained a refracture of the distal radius complicated by the bending of a previously implanted volar locking plate. To our knowledge, this is the first case reported involving the bending of a modern titanium volar locking plate, highlighting the potential for implant failure even in contemporary devices.

## Case presentation

**Figure 1:**
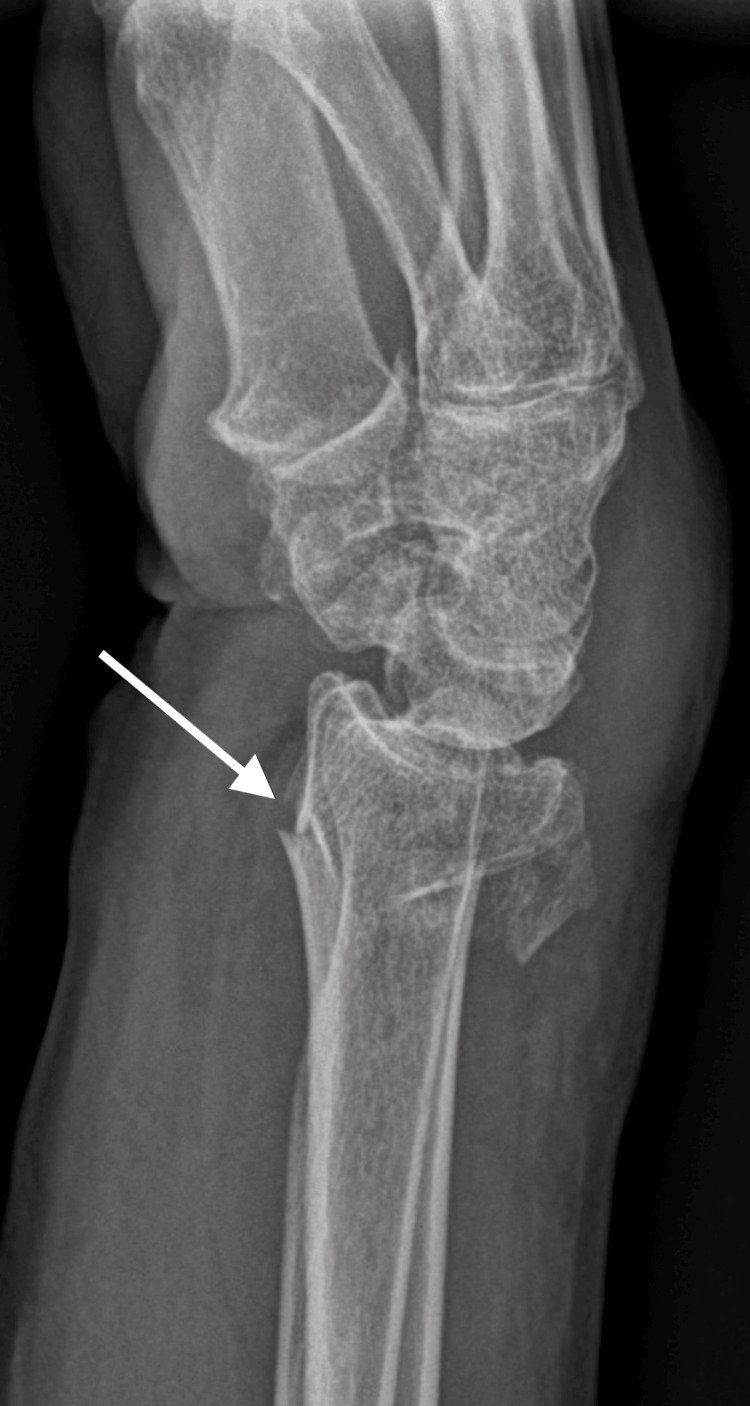
X-ray of initial fracture, lateral view

**Figure 2:**
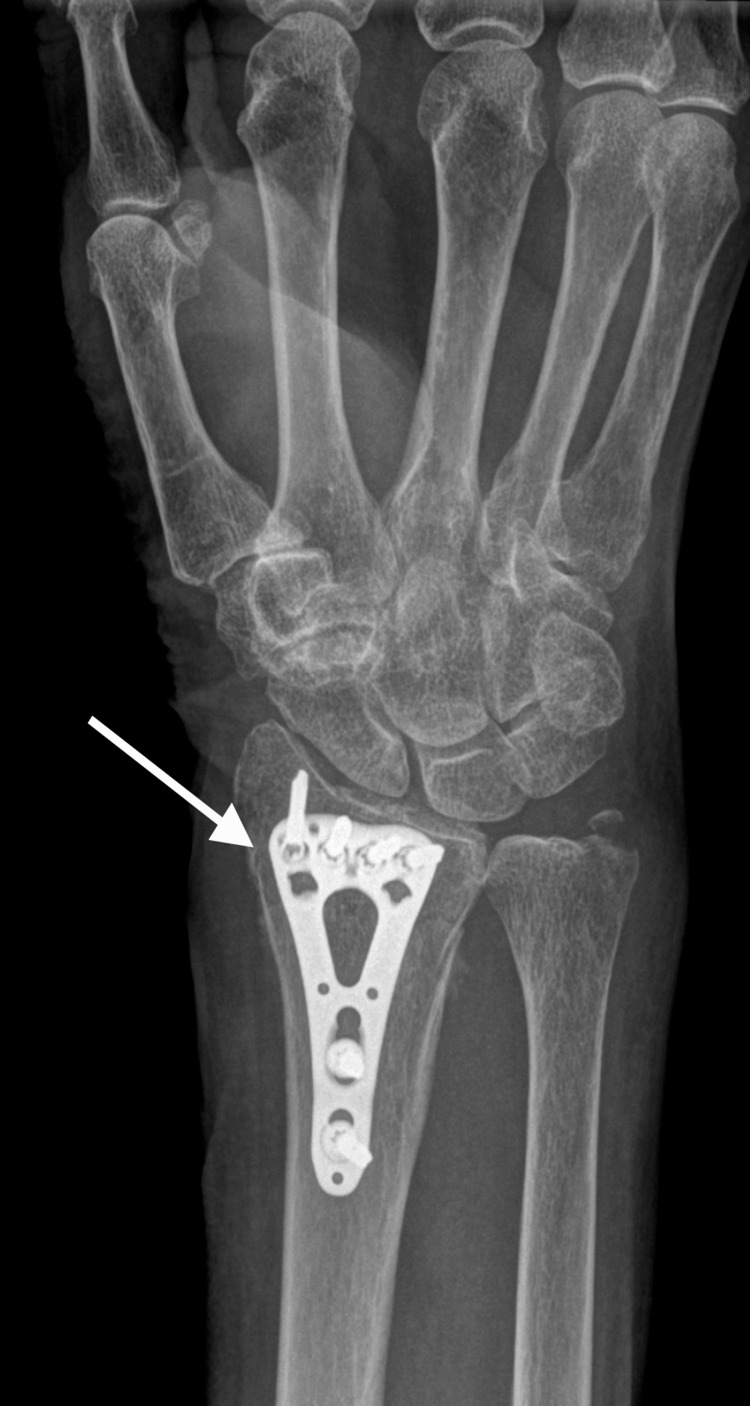
X-ray of the initial fracture at 14-month follow-up, anterior-posterior view, showing ongoing healing with encouraging signs of bridging callus formation

**Figure 3:**
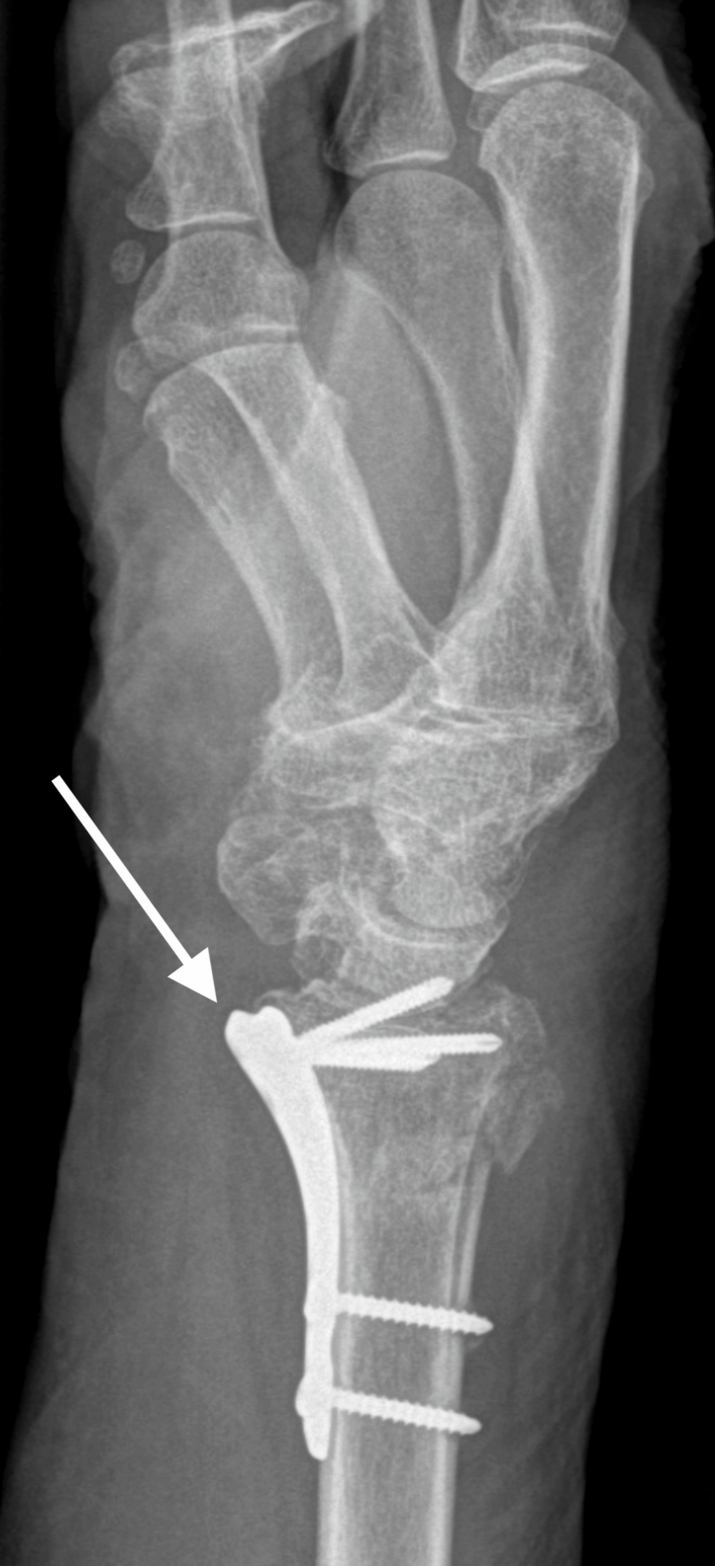
X-ray of the initial fracture at 14 months follow-up, lateral view, showing ongoing healing with encouraging signs of bridging callus formation

The patient, an 80-year-old woman, presented to the emergency department following a fall in her right hand. The patient experienced a simple fall from a standing height, resulting in a direct impact on her outstretched right hand. She attempted to break the fall by placing her right hand forward, leading to a forceful transmission of impact to the distal radius of her right wrist. The mechanism of the injury involved axial loading and hyperextension of the wrist. She had a history of a distal radius fracture treated with volar plate fixation 2.5 years ago, from which she had fully recovered (Figures 1-3).

**Figure 4:**
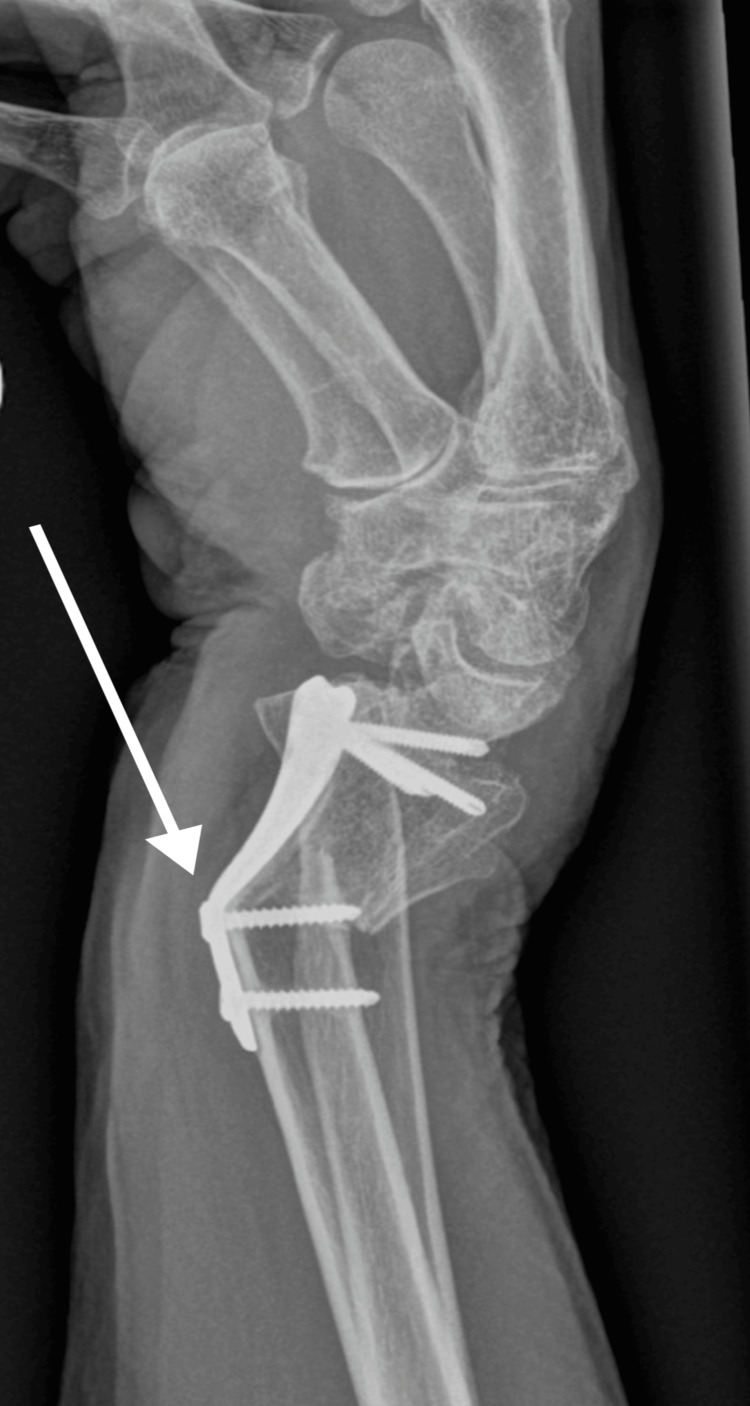
X-ray of radius refracture with bending plate, lateral view

**Figure 5:**
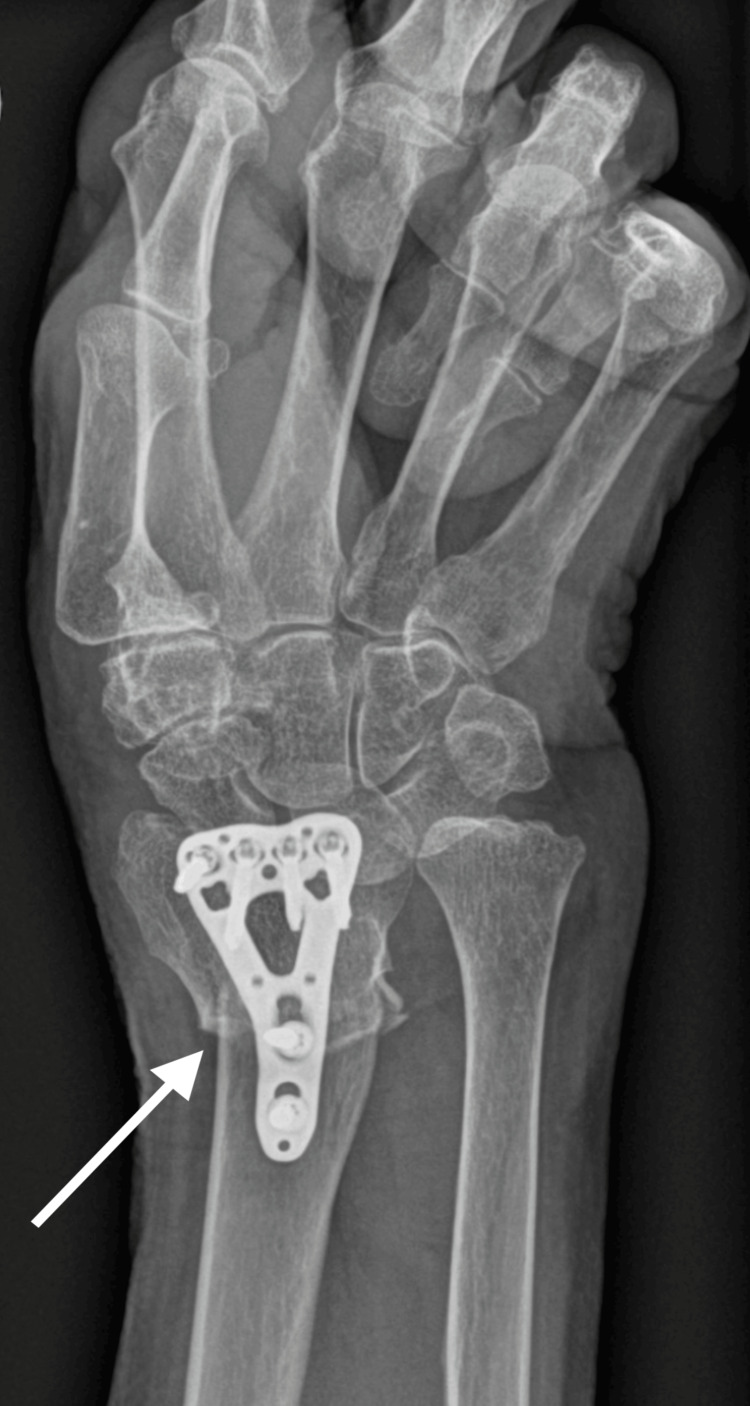
X-ray of radius refracture with bending plate, anterior posterior view

On clinical examination, the patient displayed a deformity of the right wrist without neurovascular compromise. Radiographs revealed a new distal radius fracture, located at a different site from the initial fracture, along with a bending of the pre-existing volar plate (Figures 4, 5).

**Figure 6:**
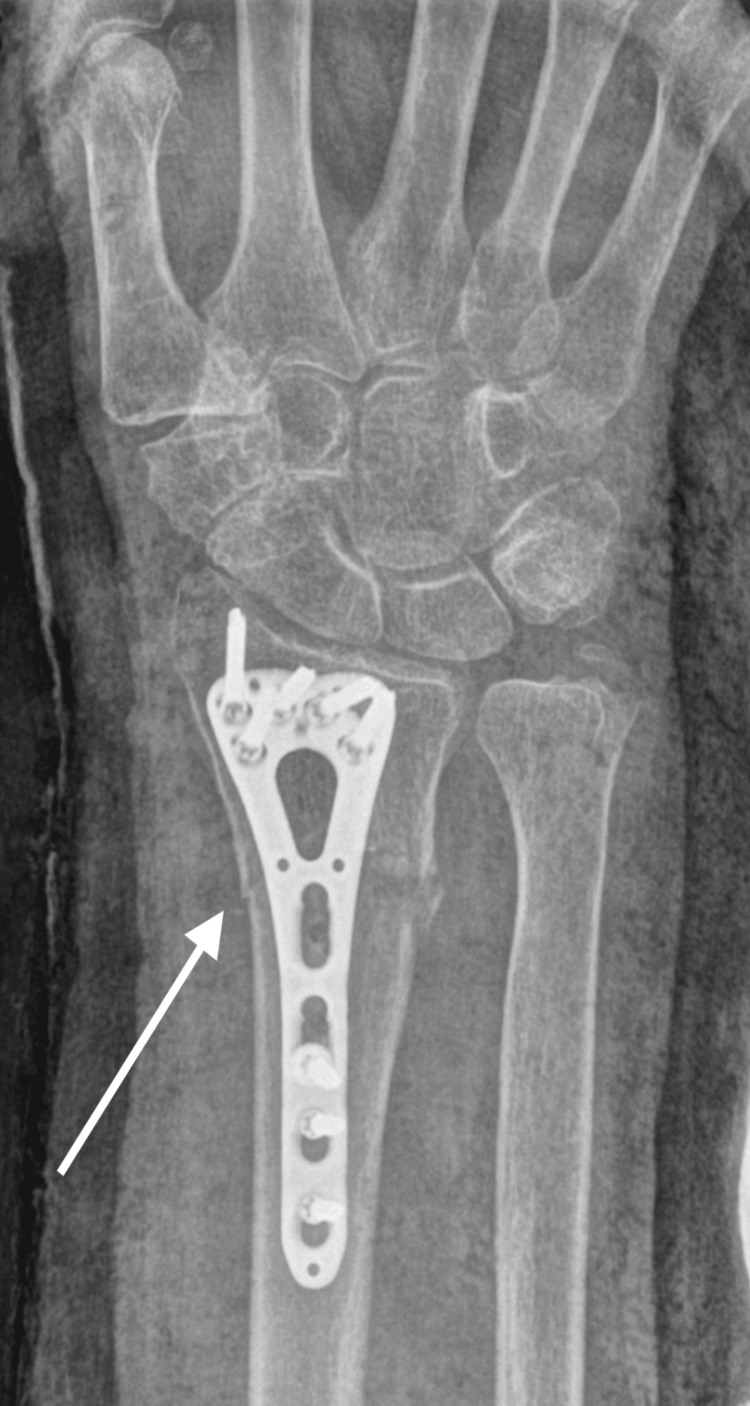
X-ray after bended plate removal and new plate insertion, anterior posterior view

**Figure 7:**
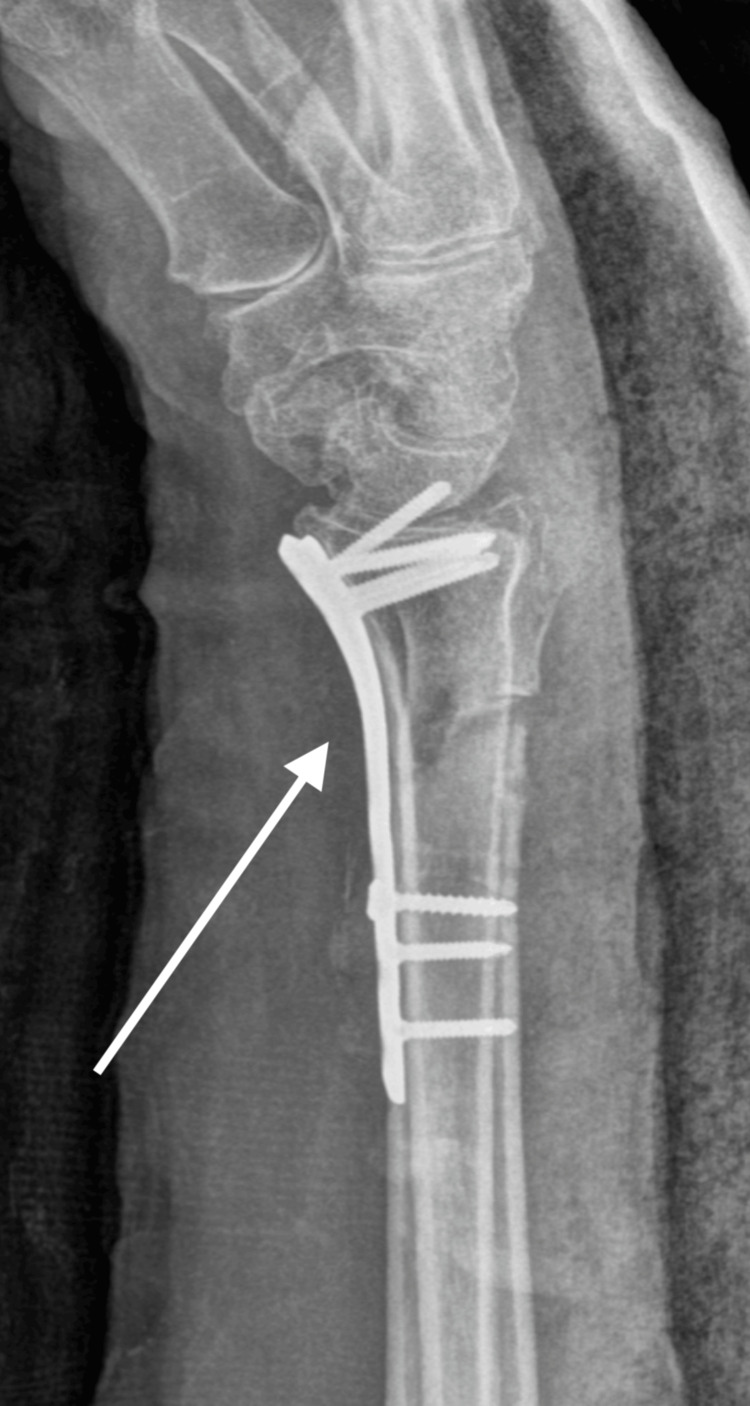
X-ray after bended plate removal and new plate insertion, lateral view

The plate had dorsally angulated by 50°, indicating significant implant failure. The patient was taken to surgery where a modified Henry approach was employed to access the fracture site. Fortunately, we encountered no significant difficulties during the approach and there was no fibrosis, and the surgical field remained clear. The removal of the bent volar plate proceeded without complication, despite the plate's dorsal angulation. With the new plate in place, there were no issues with screw fixation, and the revision surgery went smoothly, allowing for stable osteosynthesis and an uneventful recovery. The pre-existing screw holes did not present any challenges, and the new hardware was successfully positioned to ensure proper stability (Figures 6, 7).

The retrospective review of her follow-up visits confirmed that the initial fracture had healed completely and that the patient had regained full wrist function before the fall.

## Discussion

The bending of volar plates in the treatment of distal radius fractures is an exceedingly rare event, particularly with the use of modern titanium plates [2]. These early designs (stainless steel), while effective, lacked the strength and biomechanical advantages of contemporary systems [3]. Modern volar plates are now constructed from titanium, a material known for its superior strength and higher load to failure when compared to other materials [4]. Additionally, the advent of locking volar plate systems has further improved fracture fixation by providing angular stability between the plate and screws, reducing the risk of screw loosening or failure in osteoporotic bone. In this case, the refracture of the distal radius and subsequent failure of a titanium-locking volar plate presents a unique clinical challenge. The literature only reports two other cases of volar plate bending, both involving stainless steel plates from older designs. In one of these cases, the bending was accompanied by screw avulsion, necessitating surgical revision [5]. In the other case, bending occurred in a patient with an older-generation stainless steel plate, but reduction was achieved with closed management, and no further surgery was required [6]. These older stainless steel plates were more susceptible to mechanical failure under stress due to their material properties and lack of locking technology [7]. In elderly patients, implant removal is generally not indicated unless there are symptoms such as pain or functional limitations. Literature supports this conservative approach to avoid unnecessary surgical risks [8,9]. However, had the plate been removed, it might have simplified the revision surgery, avoiding the need to extract the bent implant. While international guidelines do not mandate routine hardware removal, this case highlights the importance of individualized decisions based on the patient’s risk factors and clinical presentation. Our case stands out because it involves a locking titanium volar plate, which is typically more resilient. The bending of this plate without any associated screw failure or displacement challenges the perceived biomechanical superiority of titanium locking systems, especially under the high-impact force of a fall. This raises questions about whether there are limits to the durability of modern volar plating systems, even in cases where the fracture had initially healed, and the patient had returned to normal function. Several factors could have contributed to the plate's failure in this patient, including advanced age, decreased bone quality, and the high-energy nature of the trauma. Elderly patients, particularly those with osteoporotic bone, are at a higher risk of both fracture and implant failure. Although locking volar plates are designed to provide enhanced stability in osteoporotic bone, the forces generated by a direct fall on an outstretched hand could still overwhelm the implant, leading to failure. This case highlights the importance of considering implant failure as a potential complication, even with modern plating systems, and underscores the need for further research into the biomechanical limits of locking volar plates. The findings in this case suggest that even titanium plates may not be impervious to failure under extreme mechanical stress, and close monitoring of patients with implants, particularly in elderly or osteoporotic populations, remains crucial. Additionally, this case adds valuable data to the limited body of literature on volar plate bending and may prompt further studies into optimizing implant design and material selection for high-risk patients.

## Conclusions

This case underscores a rare instance of volar plate bending in a modern titanium locking implant following a refracture of the distal radius in an elderly patient. Although volar plates are widely considered reliable and are frequently employed in fracture management due to their biomechanical advantages, this instance illustrates that implant failure can still occur, particularly in the context of high-impact trauma or compromised bone quality often seen in geriatric populations. The event highlights the importance of considering patient-specific factors, such as bone density and activity levels when selecting and positioning implants. Moreover, it calls attention to the ongoing need for research into the biomechanical limits and performance of volar plating systems. This case serves as a valuable reminder to clinicians to maintain a high index of suspicion for potential implant failure, even in the context of modern technological advances, ensuring that appropriate follow-up and intervention are prioritized when necessary.
